# Supplementary material for: Apolipoprotein A-IV-Deficient Mice in 129/SvJ Background Are Susceptible to Obesity and Glucose Intolerance
Source: Nutrients. 2023 Nov 20;15(22):4840. doi: 10.3390/nu15224840 (PMC10674380; doi:10.3390/nu15224840)

**Supplemental figures:**

**Figure S 1:** Cumulative locomotor activity was compared between apoA-IV$^{-/-}$ and WT mice at week 16. ApoA-IV$^{-/-}$ mice exhibited a significant decrease in cumulative locomotor activity at the end of a 24-hour measurement. Values are expressed as means ± SEM. $n = 11$ in WT group, $n = 7$ in apoA-IV−/− group. * $p < 0.05$, compared between the groups.

**Figure S1:**

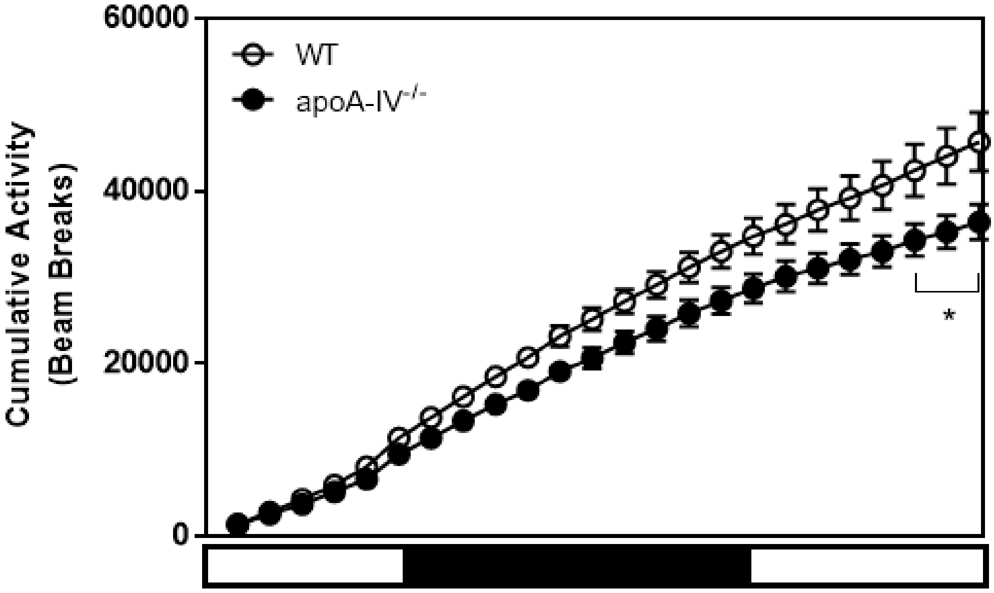

Supplement: Supplementary file 1 [file nutrients-15-04840-s001.zip › Supplementary materials 11142023.pdf]
